# Ultrasound parameters of arteries and heart in normal fetuses

**DOI:** 10.1186/s12947-024-00328-w

**Published:** 2024-07-29

**Authors:** Guihong Chen, Pin Wang, Yanhong Zhang, Na Li, Liman Fu, Yu Chen, Xuna Geng, Yongfeng Han, Lu Qin, Bulang Gao, Tianxiao Yu, Jie Mi

**Affiliations:** 1 Department of Medical Ultrasound, Research Center for Clinical Medicine Sciences, The Fourth Hospital of Shijiazhuang, No.16 Tangu North Street, Shijiazhuang, 050000 Hebei Province China; 2 Cardiology Department, Shijiazhuang People’s Hospital, No. 365, Jianhua South Street, Shijiazhuang, 050000 Hebei Province China

**Keywords:** Normal fetuses, Prenatal ultrasound parameter, Trend, gestational age, Image

## Abstract

**Background:**

Currently, no normal ultrasound data of the fetuses during the 20–40 gestation have been obtained for references of fetal growth and development. If such ultrasound data existed for prenatal diagnosis of possible diseases and abnormalities, neonates would be able to get timely treatment immediately after birth. This study was thus performed to obtain ultrasound parameters of normal fetuses during the 20–40 week gestation and the distribution of ultrasound parameters with the gestational age for references of detecting potential fetal diseases and abnormalities.

**Methods:**

Normal fetuses without any abnormalities were enrolled, and the ultrasound parameters of the general biology, arteries, and aorta were measured and analyzed.

**Results:**

417 normal fetuses were enrolled. A significant (*P* < 0.05) negative correlation with the gestational age was detected in the peak systolic velocity/peak diastolic velocity (S/D), pulsatility index (PI) and resistance index (RI) of the umbilical artery (UA). A relatively stable relationship with the gestational age was detected in the fetal weight%, S/D, PI and RI of the middle cerebral artery (MCA), peak systolic velocity (PSV) and velocity time integral (VTI) of the intra-abdominal UA, fetal heart to chest ratio, mitral valve (MV)- and tricuspid valve (TV)-E/A peak flow velocity, aortic isthmic Z-score and displacement, distance between the brachiocephalic artery-left common carotid artery (BA-LCCA) and LCCA-left subclavian artery (LSA), Z-score of aorta, ascending aorta (AAO), pulmonary artery (PA), main pulmonary artery (MPA), and descending aorta (DAO). A significant (*P* < 0.05) positive correlation with the gestational age was detected in the fetal biological data, MCA PSV and VTI, free-UA PSV and VTI and cardio-thoracic ratio, cardiac parameters, ductus arteriosus (DA) and isthmus diameter, aortic parameters, PA and MPA diameter, MPA PSV and VTI, isthmus flow volume and velocity and PA flow volume, DA and BA parameters, and LCCA and LSA parameters (flow volume, PSV, and VTI).

**Conclusion:**

A certain correlation and distribution trend is detected in the ultrasound parameters of normal fetuses, and the ratios among different parameters remain relative stable. These findings can be used for determination of abnormal growth of the fetuses in prenatal ultrasound scan.

**Graphic Abstract:**

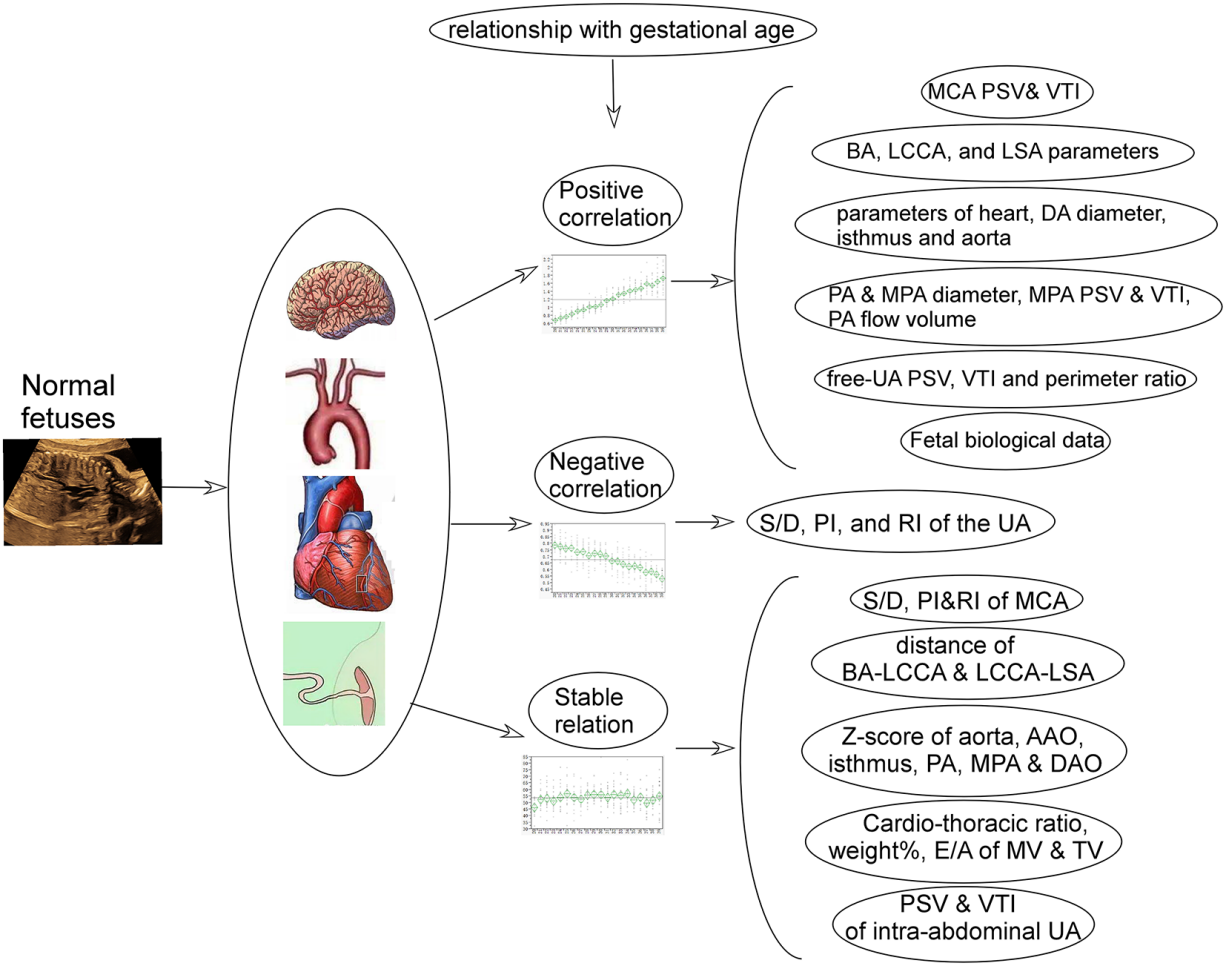

**Supplementary Information:**

The online version contains supplementary material available at 10.1186/s12947-024-00328-w.

Authors:

## Introduction

Both fetal size and growth velocity (change in size with time) have been used to assess fetal growth and development abnormalities [1–10]. Use of fetal size is common, and growth velocity evaluation is also a logical approach for fetal growth determination. For growth velocity, serial measurements of the fetuses are necessary over a large range of fetal age, however, strict serial evaluation of the fetuses may be difficult and only a limited number of studies have been conducted except for studies on individualized growth assessment [11–13]. Fetal growth velocity evaluation is focused on detection of growth abnormalities, and individualized growth assessment uses growth velocity mainly for evaluation of growth potential [14]. Measurement of the second trimester growth data can be used to specify the Rossavik size models to generate the expected third trimester size trajectories and birth features [11]. Good agreement has been achieved between the actual values and the expected data in fetuses and neonates with normal neonatal growth results as proved by the prenatal percent deviations and growth potential realization indexes in neonates [11, 14, 15]. Nonetheless, no normal ultrasound data of the fetuses during the 20–40 week gestation have been obtained for references of fetal growth and development. If such ultrasound data existed for prenatal diagnosis of possible diseases and abnormalities, neonates would be able to get timely treatment immediately after birth. This study was thus performed to obtain ultrasound parameters of normal fetuses during the 20–40 week gestation and the distribution of ultrasound parameters with the gestational age for references of detecting potential fetal diseases and abnormalities.

## Materials and methods

### Subjects

This prospective single-center study was approved by the ethics committee of the Fourth Hospital of Shijiazhuang with informed consent given by the pregnant women to participate. All methods were performed in accordance with the relevant guidelines and regulations. From May 2021 to May 2023, pregnant healthy women with antenatal sonography, regular menstruation, no genetic history, no pregnancy complications, 20–40 weeks of gestational age, and healthy neonates without abnormalities confirmed after birth were enrolled. The inclusion criteria were healthy pregnant women with known last menstrual time, no abnormalities by prenatal ultrasound screening, regular menstruation, 20–40 weeks of gestational age, antenatal sonography of the fetuses, delivery in our hospital, follow-up up to 6 months after birth, and no abnormalities confirmed by sonography and postpartum follow-up. The exclusion criteria were pregnant women with irregularity of menstruation, unknown time of last menstruation, genetic diseases, pregnancy complications, infectious diseases, and multiple pregnancies.

### Instruments and methods

**Fig. 1:**
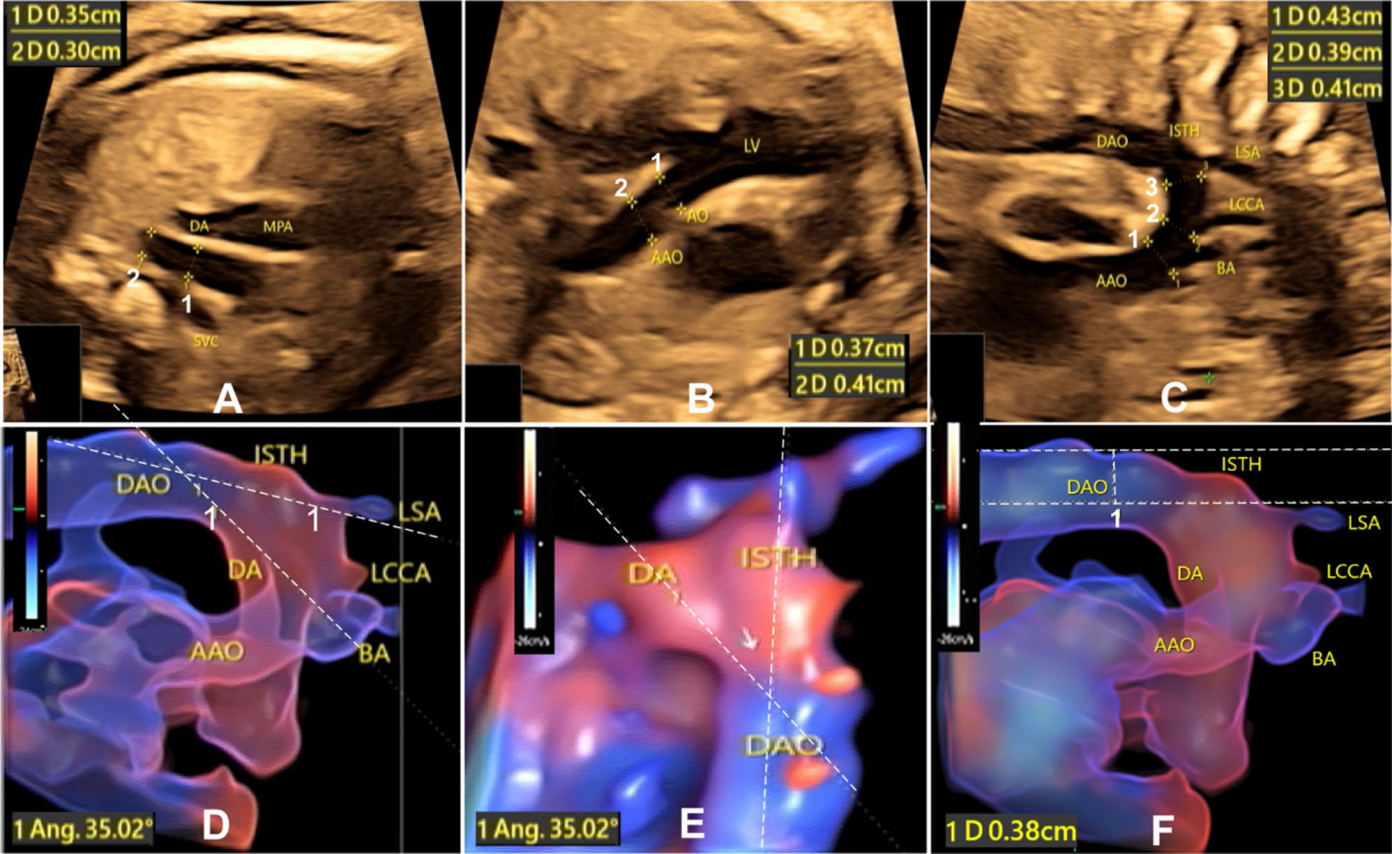
Parameters measured on ultrasound images. **A**. The diameters of the transverse aorta (1) and the aortic isthmus (2) were measured on the 3 vessel trachea view, with the transverse aorta diameter of 0.35 cm and the isthmus diameter of 0.30 cm. **B**. The diameter of the aorta (AO) at the aortic valve annulus and the ascending aorta (AAO) was measured at the left ventricular outflow tract section, with the AO diameter (1) of 0.37 cm and the AAO diameter of 0.41 cm. **C**. The diameter of the aortic arch was measured at three locations: arch 1 before the brachiocephalic artery (BA), arch 2 before the left common carotid artery (LCCA) and arch 3 before the left subclavian artery (LSA). **D**. The ductus arteriosus (DA)-isthmus angle was measured on the HD Live Flow image on the sagittal view, with the DA-Isthmus angle of 35.02°. **E**. The DA-isthmus angle was measured on the HD Live Flow image viewed from the bird’s eye’s view, with the DA-Isthmus angle of 35.02°. **F**. The displacement (or distance) from the LSA origin to the DAO outer edge were measured on the 3D STICK-HD Live Flow image. MPA, main pulmonary artery; SVC, superior vena cava; ISTH, aortic isthmus

Ultrasound scan was performed with the GE Voluson E10 Color Doppler ultrasound diagnostic instruments (General electric, Tiefenboch, AUSTRIA), quipped with 2D/3D transabdominal probes (frequency 2-5 MHz/4-8 MHz, probe model C1-6-D and RAB6-D, and power < 100 mW/cm^2^), STIC software package, and 4D View 7.0 Offline analysis. Under the fetal quiet state without influencing of breathing and movement, ultrasound scan and echocardiography were conducted to evaluate fetal growth, weight, and abnormalities. All the data were measured three times to obtain the average values by an experienced ultrasound physician with over 10 years of experience (Fig. 1).

### Ultrasound parameters

General biological data and ultrasound parameters were measured, including peak systolic velocity (PSV), pulsatility index (PI), resistance index (RI), S/D ratio (between the peak systolic velocity and peak diastolic velocity) and velocity time integral (VTI) of the middle cerebral artery (MCA) and umbilical artery (UA, both inside and outside the fetal abdomen before UA placental insertion). The heart axis, cardio-thoracic ratio (Area/Circumference), right and left atrium (end systole) and ventricle (end diastole) dimension, mitral valve A-peak flow velocity (MV-A), mitral valve E-peak flow velocity (MV-E), tricuspid valve A peak (TV-A), and tricuspid valve E peak (TV-E), were measured in the four-chamber view. Systolic aortic valve annular diameter, ascending aortic (AAO) inner diameter, aortic flow velocity and volume, pulmonary valve annular diameter, main pulmonary artery (MPA) inner diameter, and pulmonary artery (PA) flow velocity and volume were measured at the left and right ventricle outflow tract sections, with the spectrum automatic envelope to get the respective VTI and immediate heart rate. The left (LCO) and right (RCO) cardiac output, and combined cardiac output (CCO = LCO + RCO) were calculated according to the formula Q=(D/2)^2^ x 3.14 x VTI x HR (immediate heart rate). The diameter of ductus arteriosus (DA), transverse arch, and aortic isthmus was measured at the tracheal section of three vessels (3VTV). In the sagittal view of the aortic arch, the diameter of the aortic arch at three locations of arch 1–3, isthmus, descending aorta (DAO), three arch branches, the distance between the arch branches at the starting part, and the flow parameters were measured. The ratios among the parameters were calculated. The real-time 3D STIC mode was selected for 3D scanning. In the Color Render mode, the HD live flow imaging mode was applied to obtain the 3D image of the long axis of the aortic arch and catheter arch for relevant parameters (Fig. 1).

### Statistical analysis

The JMP software (10.01.2, SAS Institute, Cary, NC, USA) was used for the statistical analysis. Continuous measurement data meeting the normal distribution requirement were presented as mean and standard deviation or as median and interquartile range if not meeting the normal distribution. Categorical data were expressed as frequency and percentage. The Chi square analysis was performed for the relationship of ultrasound parameters with the gestational age, with the R^2^ and F ratio calculated. The significant P value was set at < 0.05.

## Results

417 normal fetuses without any abnormalities were enrolled, and the ultrasound parameters of the fetal general biology, MCA, UA, DA, aortic isthmus, aorta, aortic arch, arch arterial branches (brachiocephalic artery or BA, LCCA or left common carotid artery, and LSA of left subclavian artery), PA, MPA, and DAO were obtained (Tables 1, 2 and 3), including the PI, RI, VTI, PSV, S/D, diameter, isthmus displacement, Z-score, flow velocity, flow volume, LCO, CCO, and ratios between different parameters.

**Table 1:** Biological, MCA, UA, and cardiac data

Variables	Data	Variables	Data
Pregnant women no.	417	MCA/intra-UA PSV	0.26–1.93(0.70 ± 0.28)
Pregnant women age (y)	19–42 (30.2 ± 3.9)	MCA/intra-UA S/D	0.29–4.39(1.50 ± 0.54)
GA (w)	20–40 (29.76 ± 5.71)	MCA/intra-UA PI	0.61–3.75(1.56 ± 0.44)
BPD (cm)	0.84–10.2 (7.47 ± 1.52)	MCA/intra-UA RI	0.73–1.88(1.17 ± 0.19)
HC (cm)	17.03–36.01 (27.02 ± 5.1)	MCA /intra-UA VTI	0.09–1.56(0.53 ± 0.22)
AC(cm)	14.58–39.16 (25.51 ± 6.31)	MCA /free-UA PSV	0-2.24(0.96 ± 0.28)
FL (cm)	3.15–7.83 (5.58 ± 1.28)	MCA/ free-UA S/D	0.69–5.63(1.77 ± 0.54)
HL (cm)	2.95–6.89 (4.98 ± 1.02)	MCA /free-UA PI	0.65–3.25(1.74 ± 0.44)
Estimated weight (g)	49.8–4553(1687.32 ± 1087.49)	MCA/free-UA RI	0.87–2.11(1.27 ± 0.19)
Weight%	9.4–99(47.32 ± 20.78)	MCA/free-UA VTI	0.48–3.62(1.43 ± 0.43)
MCA PSV(cm/s)	17.97–94.06(43.44 ± 14.71)	cardiac axis(°)	22.75–57.84(34.83 ± 6.48)
MCA S/D	2.23–10.03(4.62 ± 1.16)	HCR	0.19–0.42(0.28 ± 0.03)
MCA PI	0.74–2.59(1.60 ± 0.30)	HC Perimeter ratio	0.45–0.59(0.53 ± 0.03)
MCA RI	0.55–1.37(0.77 ± 0.07)	Left atrium(cm)	0.59–2.01(1.17 ± 0.29)
MCA VTI(cm)	3.59–21.19(8.87 ± 3.59)	Right atrium(cm)	0.58–2.32(1.27 ± 0.35)
Intra-UA PSV(cm/s)	24.73-114.97(64.77 ± 13.45)	Right atrium/left atrium	0.80–1.47(1.09 ± 0.09)
Intra-UA S/D	1.76–10.18(3.40 ± 1.19)	LV(cm)	0.55–2.08(1.17 ± 0.31)
Intra-UA PI	0.56–1.91(1.08 ± 0.26)	RV(cm	0.57–2.23(1.18 ± 0.34)
Intra-UA RI	0.43–0.94(0.67 ± 0.10)	RV/LV	0.81–1.37(1.01 ± 0.08)
Intra-UA VTI(cm)	5.6-38.21(17.12 ± 4.24)	MV-E(cm/s)	19.37–60.08(36.77 ± 7.03)
Free-UA PSV(cm/s)	21.13–87.42(45.59 ± 9.97)	MV-A(cm/s)	31.8-84.48(53.62 ± 8.33)
Free-UA S/D	1.54–4.47(2.72 ± 0.56)	MV-E/A	0.46–1.16(0.69 ± 0.10)
Free-UA PI	0.44–1.5(0.95 ± 0.19)	TV-E(cm/s)	17.95–74.76(43.86 ± 8.76)
Free-UA RI	0.35–0.78(0.62 ± 0.08)	TV-A(cm/s)	16.42–89.18(60.50 ± 9.25)
Free-UA VTI(cm)	5.21–24.34(12.52 ± 3.45)	TV-E/A	0.50–0.93(0.72 ± 0.08)

*Note* GA, gestational age; BPD, biparietal diameter; HC, head circumference; AC, abdomen circumference; FL, femur length; HL, humerus length; MCA, middle cerebral artery; PSV, peak systolic velocity; S/D, PSV/EDV (end diastolic velocity); PI, pulsatility index; RI, resistance index; VTI, velocity time integral; UA, umbilical artery; Intra- UA, the UA segment inside the fetal abdomen; free-UA, the UA segment outside the fetus before the UA placental insertion; HCR, Heart to chest area ratio; HC, Heart to chest; RV, Right ventricle; LV, left ventricle; MV-E, Mitral valve E-peak flow velocity; MV-A, mitral valve A-peak flow velocity; TV-E, tricuspid valve E peak; TV-A, tricuspid valve A peak. Data were presented as mean and standard deviation if in the normal distribution. Data were presented as mean ± standard deviation if meeting the normal distribution or as median and interquartile range if not in the normal distribution

**Table 2:** Data of DA, isthmus, aorta, aortic arch, arch arteries, and pulmonary artery

Variables	Data	Variables	Data
DA diameter (cm)	0.16–0.76(0.37 ± 0.14)	LCCA-LSA/arch 3 diameter	0.09–1.64(0.55 ± 0.26)
3VTV Isthmus diameter(cm)	0.18–0.59(0.35 ± 0.09)	LCCA-LSA/BA-LCCA	0.36–7.6(1.71 ± 0.81)
SV Isthmus diameter(cm)	0.15–0.55(0.32 ± 0.08)	AO diameter(cm)	0.21–0.79(0.44 ± 0.12)
SV Isthmus ZS	-6.03- 2.65(-1.21, 0.49)	AO-ZS	-2.81-1.25(-0.76,0.83)
3VTV Isthmus/DA	0.52–1.52(0.99 ± 0.16)	AAO(cm)	0.22–0.8(0.47 ± 0.13)
SV Isthmus/DA	0.42–1.52(0.91 ± 0.17)	AAO-ZS	-3.44-0.68(-1.26 ± 0.62)
3VTV Transverse arch diameter(cm)	0.19–0.73(0.42 ± 0.12)	AO flow velocity(cm/s)	56.31-136.78(91.86 ± 13.58)
Arch diameter 1(cm)	0.12–0.82(0.47 ± 0.12)	AO VTI(cm)	8.03–19.52(13.29 ± 1.95)
Arch diameter 2(cm)	0.09–0.68(0.36 ± 0.10)	AO flow volume (ml/min)	49.63-1170.47(306.86, 288.5)
Arch diameter 3(cm)	0.09–0.67(0.35 ± 0.09)	AO flow/weight(ml/ min/g)	0.11–9.79(0.21,0.06)
Arch diameter 3/2	0.68–1.5(0.96 ± 0.08)	PA (cm)	0.28–0.99(0.54 ± 0.15)
SV Isthmus /arch 1 diameter	0.48–1.67(0.69 ± 0.10)	PA-ZS	-3.23-0.97(-0.92,0.87)
SV isthmus /arch 2 diameter	0.63–2.22(0.90 ± 0.14)	MPA(cm)	0.33–1.32(0.73 ± 0.22)
SV Isthmus /arch diameter 3	0.65–2.22(0.93 ± 0.13)	MPA-ZS	-1.92-2.37(0.74, 0.91)
SV isthmus diameter/AAD	0.47–0.92(0.69 ± 0.07)	MPA PSV(cm/s)	42.38–99.79(68.60 ± 10.84)
SV isthmus diameter/AD	0.47-1(0.73 ± 0.08)	PA VTI(cm)	6.72–17.33(11.47 ± 2.02)
3D LSA-DAO displacement (cm)	0.09–34.5(0.39 ± 1.68)	PA flow volume(ml/min)	65.57-1344.22(377.52,430)
3D STIC Isthmus/DA angle (°)	0.33–68.32(24.44 ± 9.96)	PA flow volume/ weight (ml/min /g)	0.13–14.51(0.30 ± 0.71)
3D HD live flow DA-Isthmus angle (above view) (°)	8.45–79.86(27.82 ± 8.36)	AO flow /PA flow	0.01–1.34(0.81 ± 0.16)
3D HD live flow DA- Isthmus (SV)(°)	6.65–68.63(24.52 ± 10.02)	CCO(ml/min)	115.77-2514.69(674.72, 702)
BA(cm)	0.09–0.49(0.24 ± 0.08)	CCO/weight(ml/min/g)	0.25–24.32(0.47,0.11)
LCCA(cm)	0.09–0.46(0.19 ± 0.07)	PA/AAO	0.91–1.45(1.15 ± 0.09)
LSA(cm)	0.06–0.37(0.19 ± 0.06)	PA/AO	1-13.42(1.24 ± 0.60)
BA-LCCA distance(cm)	0.04–0.34(0.12 ± 0.05)	MPA/AAO	0.95–2.06(1.55 ± 0.18)
LCCA-LSA distance(cm)	0.04–0.52(0.18 ± 0.09)		

*Note* DA, ductus arteriosus; 3VTV, 3 vessel trachea view; SV, sagittal view; ZS, Z-score; isthmus, aortic isthmus; AAD, ascending aortic inner diameter; AD, aortic inner diameter; 3D, HD live flow image; DAO, descending aorta; LSA-DAO, left subclavian artery-descending aorta; BA, brachiocephalic artery trunk; LCCA, left common carotid artery; AO, aorta; AAO, ascending aorta; VTI, velocity time integral; PA, pulmonary artery; MPA, main pulmonary artery; PSV, peak systolic velocity; CCO, combined cardiac output; AO flow volume indicates the left cardiac output; PA flow volume indicates the right cardiac output. Data were presented as mean ± standard deviation if meeting the normal distribution or as median and interquartile range if not in the normal distribution

**Table 3:** Data of cardiac output, PA, aorta, isthmus, and DA

Variables	Data	Variables	Data
3VTV Isthmus/ DAO	0.53–0.96(0.76 ± 0.08)	Isthmus diastolic velocity(cm/s)	7.22–66.82(23.47 ± 8.05)
SV Isthmus/ DAO	0.44–0.96(0.70 ± 0.08)	Isthmus diastolic VTI(cm)	0.77–7.2(3.39 ± 0.89)
SV Isthmus /DA	0.42–1.52(0.91 ± 0.17)	Isthmus Diastolic flow volume(ml/min)	3.21-206.45(43.76 ± 27.17)
3VTV Isthmus/ DA	0.52–1.52(0.99 ± 0.16)	Isthmus Diastolic flow/weight(ml/min/g)	0.003-1.60(0.03, 0.13)
DAO-ZS	-2.32-2.75(-0.25, 0.88)	Isthmus diastolic /systolic VTI	0.14–1.26(0.37 ± 0.09)
DAO diameter(cm)	0.23–0.84(0.47 ± 0.13)	Isthmus diastolic /isthmus total VTI	0.12–0.53(0.27 ± 0.04)
DAO velocity(cm/s)	41.8-180.11(99.88 ± 25.42)	Isthmus/AO flow	0.15–1.12(0.51 ± 0.13)
DAO VTI(cm)	6.29–29.69(16.43 ± 4.10)	Isthmus flow/CCO	0.003–0.43(0.23 ± 0.06)
DAO flow volume(ml/min)	46.68-1826.69(397.14, 483.31)	DA PSV(cm/s)	40.92-163.11(99.73 ± 22.11)
DAO flow /weight(ml/min/g)	0.12–25.40(0.27, 0.07)	DA VTI(cm)	6.46–33.14(15.04 ± 3.57)
Isthmus PSV (Total)(cm/s)	32.32-139.71(87.22 ± 16.49)	DA flow(ml/min)	30.22-1268.88(199.17,330.12)
Isthmus VTI(cm)	5.58–20.03(12.63 ± 2.46)	DA flow/weight(ml/min/g)	0.05–7.81(0.15, 0.07)
Isthmus flow volume(ml/min)	15.70-524.94(141.47, 145.15)	BA-PSV(cm/s)	42.72-296.51(102.46 ± 31.33)
Isthmus flow volume/weight(ml/min/g)	0.02–6.41(0.11, 0.04)	BA VTI(cm)	1.73–41.98(11.85 ± 4.68)
Isthmus flow /LCO	0.15–1.12(0.51 ± 0.13)	BA flow(ml/min)	0-501.93(69.63, 104.22)
Isthmus flow volume/CCO	0.003–0.43(0.23 ± 0.06)	BA flow/weight(ml/min/g)	0-3.83(0.05, 0.03)
Isthmus/DA flow volume	0.08–2.06(0.75 ± 0.34)	LCCA PSV(cm/s)	28.95–333.5(98.83 ± 33.47)
Isthmus/DAO flow volume	0.07–0.89(0.39 ± 0.13)	LCCA VTI(cm)	3.05–47.19(11.85 ± 4.85)
Isthmus/BA flow volume	0.33–8.07(2.34 ± 1.21)	LCCA flow(ml/min)	0-292.08(45.67,78.42)
Isthmus/LCCA flow volume	0.48–14.14(3.22, 2.30)	LCCA flow/weight(ml/min/g)	0-2.85(0.03, 0.02)
Isthmus/LSA flow volume	0.66–14.27(3.89 ± 2.12)	LSA PSV(cm/s)	26.38-228.48(98.20 ± 28.78)
Isthmus systolic velocity(cm/s)	32.32-141.49(89.70 ± 16.59)	LSA VTI(cm)	3.74–26.26(11.27 ± 3.52)
Isthmus systolic VTI(cm)	4.84–15.08(9.40 ± 1.95)	LSA flow(ml/min)	0-243.34(43.89, 61.62)
Isthmus Systolic flow volume(ml/min)	13.62-338.66(120.48 ± 69.37)	LSA flow/weight(ml/min/g)	0-2.04(0.03, 0.01)
Isthmus systolic flow volume/weight(ml/min/g)	0.01–5.58(0.08, 0.03)	LSA/isthmus flow	0-1.52(0.33 ± 0.16)

*Note* PA, pulmonary artery; DA, ductus arteriosus; 3VTV, 3 vessel trachea view; DAO, descending aorta; SV, sagittal view; ZS, Z score; VTI, velocity time integral; PSV, peak systolic velocity; LCO, left cardiac output; CCO, combined cardiac output; BA, brachiocephalic artery trunk; LCCA, left common carotid artery; LSA, left subclavian artery; AO, aorta; MPA, main pulmonary artery; AAO, ascending aorta; DAO, descending aorta; LSA, left subclavian artery; VTI, velocity time integral; PSV, peak systolic velocity. Data were presented as mean ± standard deviation if meeting the normal distribution or as median and interquartile range if not in the normal distribution

**Fig. 2:**
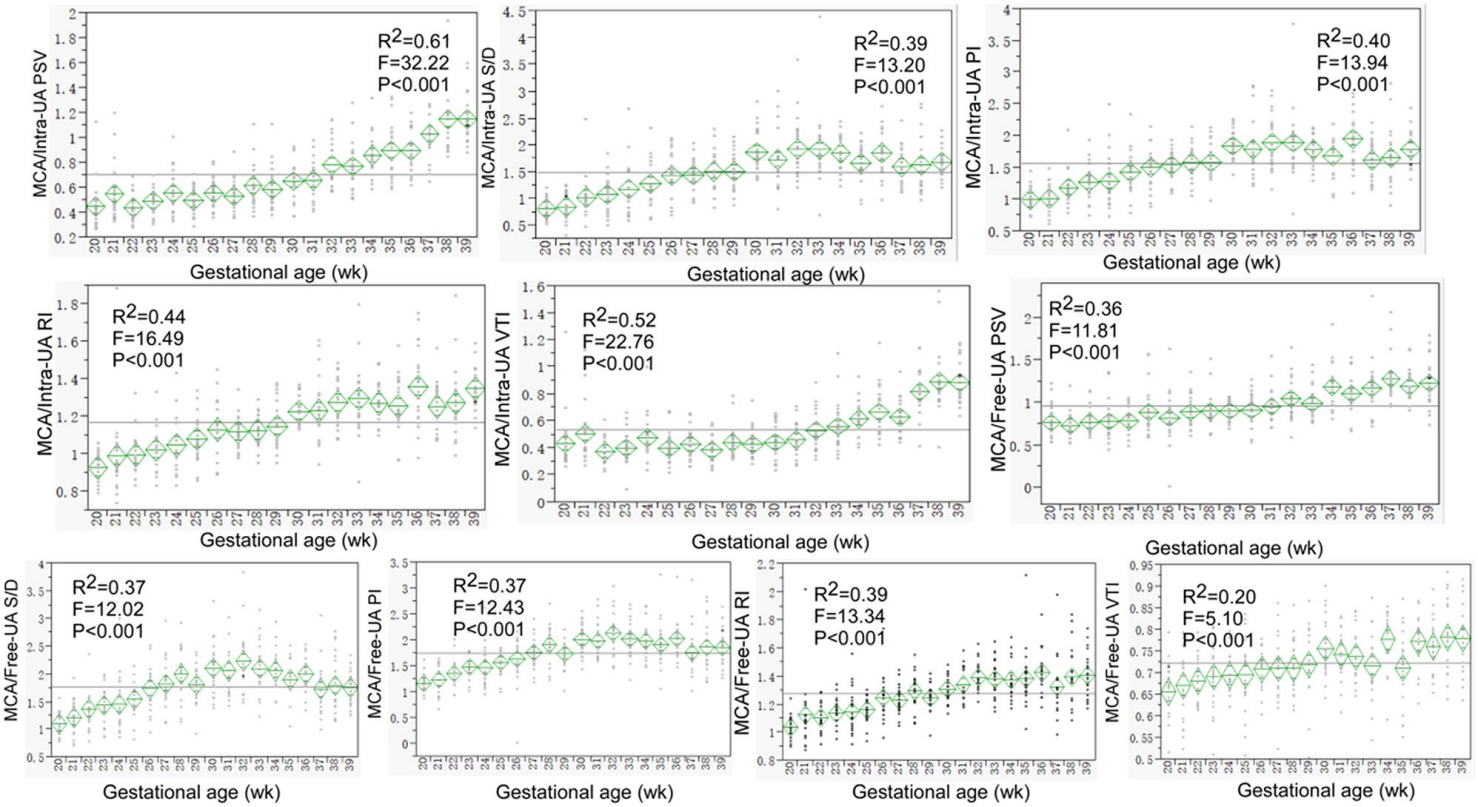
Trends of the ratios of the fetal MCA (middle cerebral artery) parameters to those of the intra-abdominal and free UA (umbilical artery) were shown with the gestational age. These ratios were in a narrow relatively-stable range

**Fig. 3:**
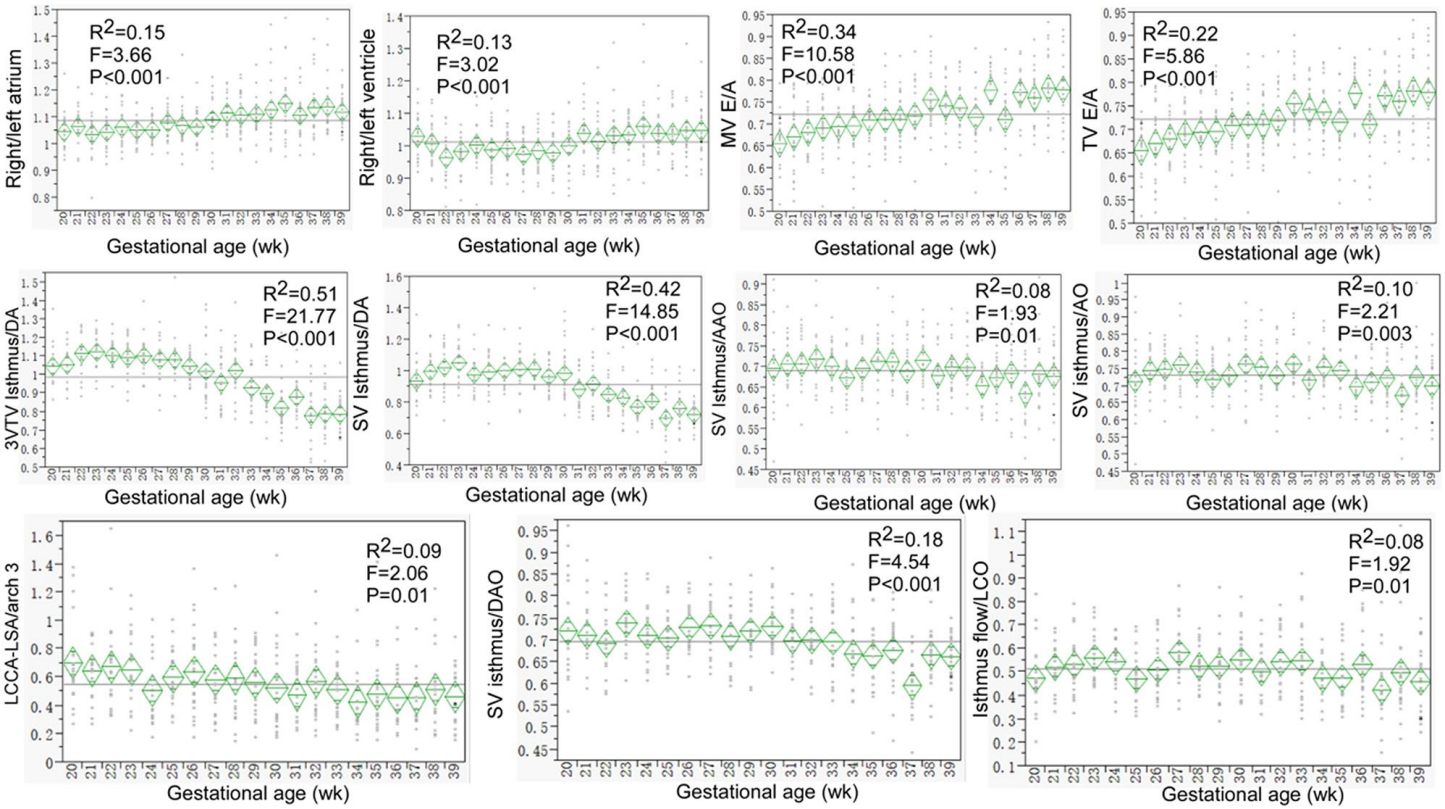
The trends with the gestational age were demonstrated in the ratios of between the right and left atrium and ventricle, MV (mitral valve)- and TV (tricuspid valve)-E/A peak flow velocity, ratio of isthmus to ductus arteriosus (DA), aorta, AAO and DAO diameter, ratio of LCCA-LSA distance to arch 3 diameter, and isthmus flow volume to the left cardiac output (LCO). These ratios were relatively stable

**Fig. 4:**
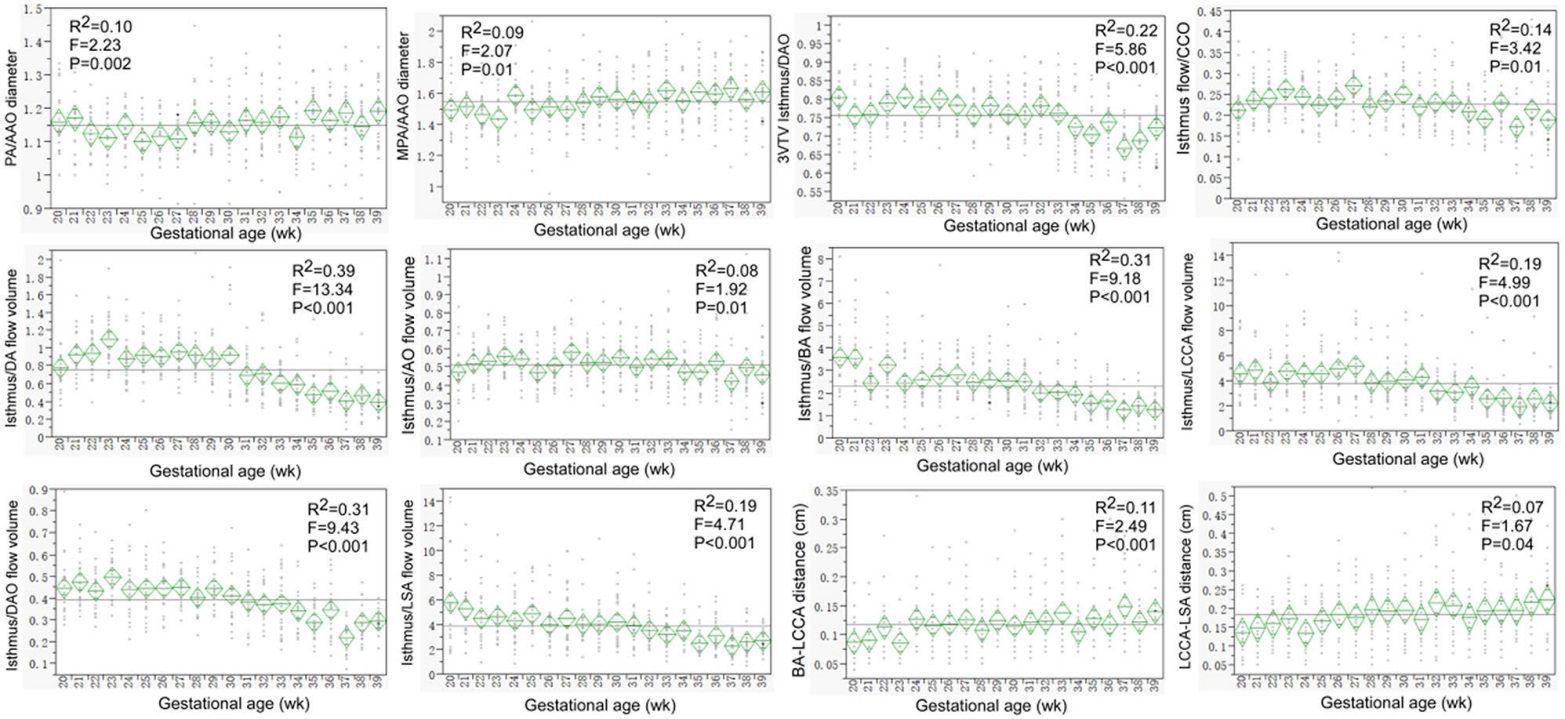
Relative stable trends with the gestational age were demonstrated in the ratios of the pulmonary artery (PA) and main pulmonary artery (MPA) to the ascending aorta (AAO) and descending aorta (DAO) diameter, of the isthmus flow volume to that of left cardiac output (LCO), combined cardiac output (CCO), ductus arteriosus (DA), aorta (AO), brachiocephalic artery (BA), left common carotid artery (LCCA), DAO, and left subclavian artery (LSA), and the BA-LCCA or LCCA-LSA distance

Most of the ultrasound parameters were significantly (*P* < 0.05) correlated with the gestational age (Figs. 2, 3 and 4 and supplemental Figs. 1–8). The ratios of the MCA data to those of the UA both inside and outside the fetal abdomen, of the right to the left atrium or ventricle, and of the isthmus diameter and flow volume to those of the DA, aorta, AAO, DAO, and arch branches remained relatively stable (Figs. 1, 2 and 3). A significant (*P* < 0.05) negative correlation with the gestational age was detected in the intra-abdominal and free UA parameters (S/D, PI and RI) (Supplemental Fig. 1). Although significant (*P* < 0.05), a relatively stable correlation with the gestational age was detected in the parameters of fetal weight%, MCA S/D, PI and RI (Supplemental Fig. 1), intra-abdominal UA PSV and VTI, fetal cardio-thoracic ratio (Supplemental Fig. 2), MV- and TV-E/A (Supplemental Fig. 3), isthmus Z-score and displacement (Supplemental Fig. 4), BA-LCCA and LCCA-LSA distance, Z-score of aorta, AAO, PA, MPA, and DAO (Supplemental Fig. 5).

A significant (*P* < 0.05) positive correlation with the gestational age was detected in the fetal biological data (Supplemental Fig. 1), MCA PSV and VTI (Supplemental Fig. 1), free-UA PSV, VTI and perimeter ratio (Supplemental Fig. 2), left and right atrium and ventricle, MV- and TV-E, TV-A, DA and isthmus diameter (Supplemental Figs. 3 and 4), aortic arch and arch branch diameter (Supplemental Fig. 4), aortic diameter and flow volume and velocity, AAO diameter and VTI, PA and MPA diameter (Supplemental Fig. 5), MPA PSV and VTI, DAO parameters (diameter, flow velocity and volume, VTI, and PSV) (Supplemental Fig. 6), isthmus flow volume and velocity, PA flow volume, DA and BA parameters (flow volume, PSV and VTI) (Supplemental Figs. 7 and 8), LCCA and LSA parameters (flow volume, PSV, and VTI) (Supplemental Fig. 8).

**Fig. 5:**
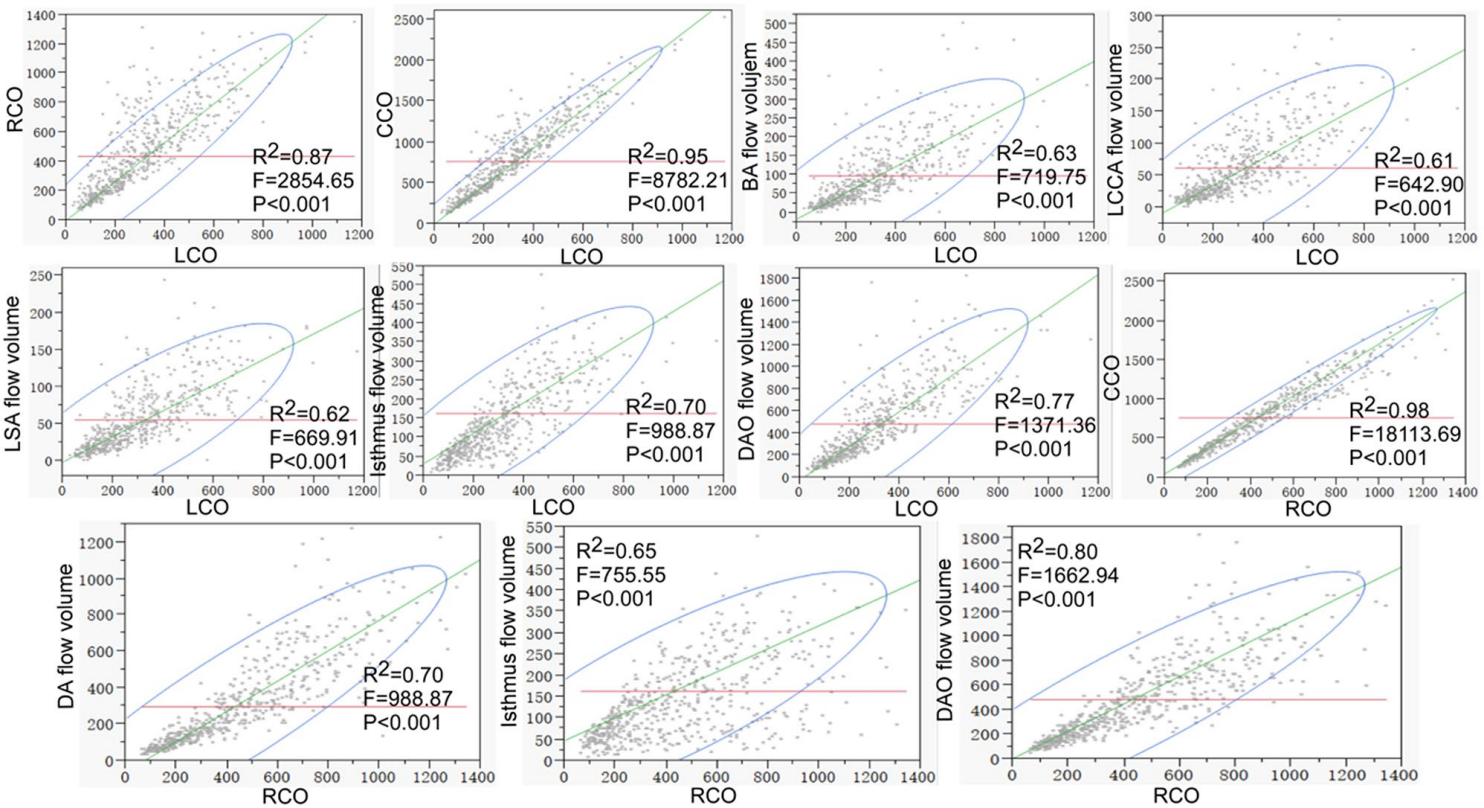
The left cardiac output (LCO) was in a significant (*P* < 0.05) positive correlation with right cardiac output (RCO), combined cardiac output (CCO), and the flow volume of brachiocephalic artery (BA), left common carotid artery (LCCA), left subclavian artery (LSA), isthmus, and descending aorta (DAO), and the RCO was significantly positively correlated with the CCO and the flow volume of ductus arteriosus (DA) and DAO. In normal condition, the RCO contributed more to the CCO and DAO flow volume, whereas the LCO contributed more to the isthmus flow volume

The right atrium and ventricle were significantly (*P* < 0.05) greater than the left atrium (1.27 ± 0.35 vs. 1.17 ± 0.29 cm, *P* < 0.001) and ventricle (1.18 ± 0.34 vs. 1.17 ± 0.31 cm, *P* = 0.001), respectively (Table 1). The PA/AAO diameter ratio was 0.91–1.45(1.15 ± 0.09). The LCO (AO flow volume) was significantly smaller than the RCO (PA flow volume) (329.37 ± 193.87 ml vs. 430.46 ± 275.39 ml, *P* < 0.001). The LCO was in a significant (*P* < 0.05) positive correlation with RCO, CCO, and the flow volume of BA, LCCA, LSA, isthmus, and DAO, whereas the RCO was significantly positively correlated with the CCO and the flow volume of DA and DAO (Fig. 5). In normal condition, the RCO contributed more to the CCO and DAO flow volume, whereas the LCO contributed more to the isthmus flow volume.

## Discussion

In this study exploring the normal range and distribution trend of ultrasound parameters with the gestational age in normal fetuses, a certain correlation was found in the ultrasound parameters of normal fetuses with the gestational age, and the ratios among different parameters remained relative stable. These findings could be used for determination of abnormal growth of the fetuses in prenatal ultrasound scan.

In fetuses with prenatal restricted growth, blood flow is redistributed from the peripheral tissues and organs to the brain, and Doppler ultrasound examination of the UA and fetal cerebral arteries (MCA in particular) can be applied to evaluate the relevant changes [16]. In these fetuses, the UA PI is increased while the MCA PI is decreased. The nomograms of the MCA PI and PSV with advanced gestational age have been reported, and decreased MCA PI has been reported in fetuses with congenital heart disease or at risk of perinatal mortality and morbidity [16–19]. Lower MCA PI and cerebral placental ratio (MCA PI/UA PI) and increased UA PI were reported in fetal hypoplastic left heart syndrome and isolated coarctation of the aorta (CoA), and the MCA PI is associated with changed fetal cerebral blood flow and positively correlates with head development [20]. The MCA PSV has been found to increase as a good indicator for perinatal mortality in a group of fetuses with restricted growth [16, 21]. Our study provided the ultrasound parameters of fetal biology, MCA and UA both inside and outside the fetal abdomen of normal fetuses as references to diagnose potential abnormalities of fetal growth. The fetal biparietal diameter, head circumference, abdomenal circumference, femur length, humerus length, estimated body weight positively correlated with the gestational age, and the ratios of the MCA data to those of the UA both inside and outside the fetal abdomen remained relatively stable during the 20–40 week gestaion investigated in our study.

The Prenatal Growth Assessment Score (PGAS) has been developed to detect different kinds of fetal growth issues except those of tissue abnormalities [22], and the PGAS was based on the head circumference, abdominal circumference, femur diaphysis length, mid-thigh circumference, and estimated weight. Nonetheless, this score is limited by reference ranges determined from all the 20–40 week gestation data without considering age-specific differences and was initially proposed for only one combination of anatomical parameters [22–24]. Our study provided the reference ranges of the fetuses during the 20–40 weeks and could be used as references for normal growth.

In fetuses suspected of CoA, the right heart structures (atrium and ventricle) are larger than the left ones, the TV is larger than the MV, and the PA/AAO diameter ratio is greater than 1.60 at the 3VTV view, with the presence of a narrowed aortic isthmus, hypoplastic transverse aortic arch, and CoA shelf [25–27]. Transverse aortic arch hypoplasia and elevated PA valve and PA diameters have also been reported in neonates with CoA, which may suggest reduced aortic arch flow and raised PA and DA flow in fetuses [28]. In our study, normal fetuses showed greater right heart structures than the left ones, but a smaller ratio of the PA/AAO diameter (range 0.91–1.45 and mean 1.15 ± 0.09), with no presence of CoA shelf, hypoplastic transverse aortic arch, or a narrowed isthmus. The right heart structures are larger than the left ones because the major blood flow of the fetus comes from the right heart. A prenatal definitive diagnosis of congenital fetal diseases like CoA can influence and decrease the mortality rate [26, 29]. Definition of the normal ultrasound parameters is helpful for detection of possible congenital fetal diseases. Antenatal ultrasound parameters have been applied for the diagnosis of CoA [30–35], including diameter of the PA or the aorta at the aortic valve, aortic isthmus, and AAO, and a ratio between the DA and aortic isthmus. The Z-scores of the aortic isthmus and AAO had been reported to be significantly lower in neonates with CoA [36, 37]. The MPA and TV Z-scores significantly rose while the mean Z-scores of the MV and the aortic isthmus significantly dropped in CoA fetuses [30]. A significant increase in the LCCA-LSA distance and aortic arch-DAO angle was also reported in CoA positive fetuses [38]. In our study, these ultrasound parameters were investigated in normal fetuses and can be used as references for detection of potential diseases.

Cardiac remodeling manifests as alterations in mass, size, geometry, and function of the heart in response to injury or load [9, 39]. This process can cause impairment in the cardiac ejection and/or relaxation ability as clinical or subclinical cardiac dysfunction. Cardiac structure dysfunction and remodeling have been described in some antenatal conditions but are scarcely described in fetuses with congenital heart diseases [9, 40]. With the cardiac ultrasound parameters obtained in normal fetuses, correct prenatal diagnosis of some congenital heart diseases can be reached earlier. In normal fetuses, the left ventricle develops under a low volume load and normal pressure condition, and after birth, the left ventricle changes to a globular shape and the filling velocities are increased because of elevated pulmonary venous return and closed foramen ovale to support the system flow. This is why the left cardiac structures, including the atrium and ventricle, were significantly smaller than the right ones in the normal fetuses as revealed by our study. This significant difference between the right and left cardiac structures may require to reach a certain degree for the diagnosis of a diseased status. Our study also found that the LCO was significantly smaller than the RCO (329.37 ± 193.87 ml vs. 430.46 ± 275.39 ml, *P* < 0.001), with the RCO contributing more to the CCO and DAO flow volume while the LCO contributing more to the isthmus flow volume.

This study had some limitations such as a relatively small cohort of fetuses, Chinese subjects enrolled only, one-center study design, and no control, which may all result in publication bias and affect the generalization of the outcome. Future prospective, multi-center studies with a lot of fetuses enrolled and multiple races and ethnicities involved will have to be conducted for better outcomes.

## Conclusion

In summary, a certain correlation exists in the ultrasound parameters of normal fetuses with the gestational age, and the ratios among different parameters remain relative stable. The right heart structures are significantly larger than the left ones in normal fetuses, with the right cardiac output contributing more to the CCO and DAO flow volume while the left cardiac output contributing more to the isthmus flow volume. These findings can be used for determination of abnormal growth of the fetuses in prenatal ultrasound scan.

### Electronic supplementary material

Below is the link to the electronic supplementary material.

Supplementary Material 1

## Data Availability

The datasets generated and/or analyzed during the current study are not publicly available due to the restriction by the hospital policy but are available from the corresponding author on reasonable request.

## Abbreviations

2D: Two dimension
3D: Three dimension
PSV: Peak systolic velocity
PI: Pulsatility index
RI: Resistance index
S/D: Peak systolic velocity/peak diastolic velocity
VTI: Velocity time integral
MCA: Middle cerebral artery
UA: Umbilical artery
MV-A: Mitral valve A peak flow velocity
TV-E: Tricuspid valve E peak flow velocity
AO: Aorta
AAO: Ascending aorta
MPA: Main pulmonary artery
PA: Pulmonary artery
LCO: Left cardiac output
RCO: Right cardiac output
CCO: Combined cardiac output
DA: Ductus arteriosus
3VTV: 3 vessel tracheal view
DAO: Descending aorta
LCCA: Left common carotid artery
BA: Brachiocephalic artery
LSA: Left subclavian artery
CoA: Coarctation of the aorta
PGAS: Prenatal growth assessment score
GA: Gestational age
BPD: Biparietal diameter
HC: Head circumference
AC: Abdomen circumference
FL: Femur length
HL: Humerus length
SV: Sagittal view
ZS: Z-score
AAD: Ascending aortic inner diameter
AD: Aortic inner diameter
SVC: Superior vena cava
ISTH: Aortic isthmus
