# Supplementary material for: Ultrasound parameters of arteries and heart in normal fetuses
Source: Cardiovasc Ultrasound. 2024 Jul 29;22:9. doi: 10.1186/s12947-024-00328-w (PMC11285228; doi:10.1186/s12947-024-00328-w)
Supplement: Supplementary file 1 — Supplementary Material 1 [file 12947_2024_328_MOESM1_ESM.docx]

Supplemental figures and figure legends


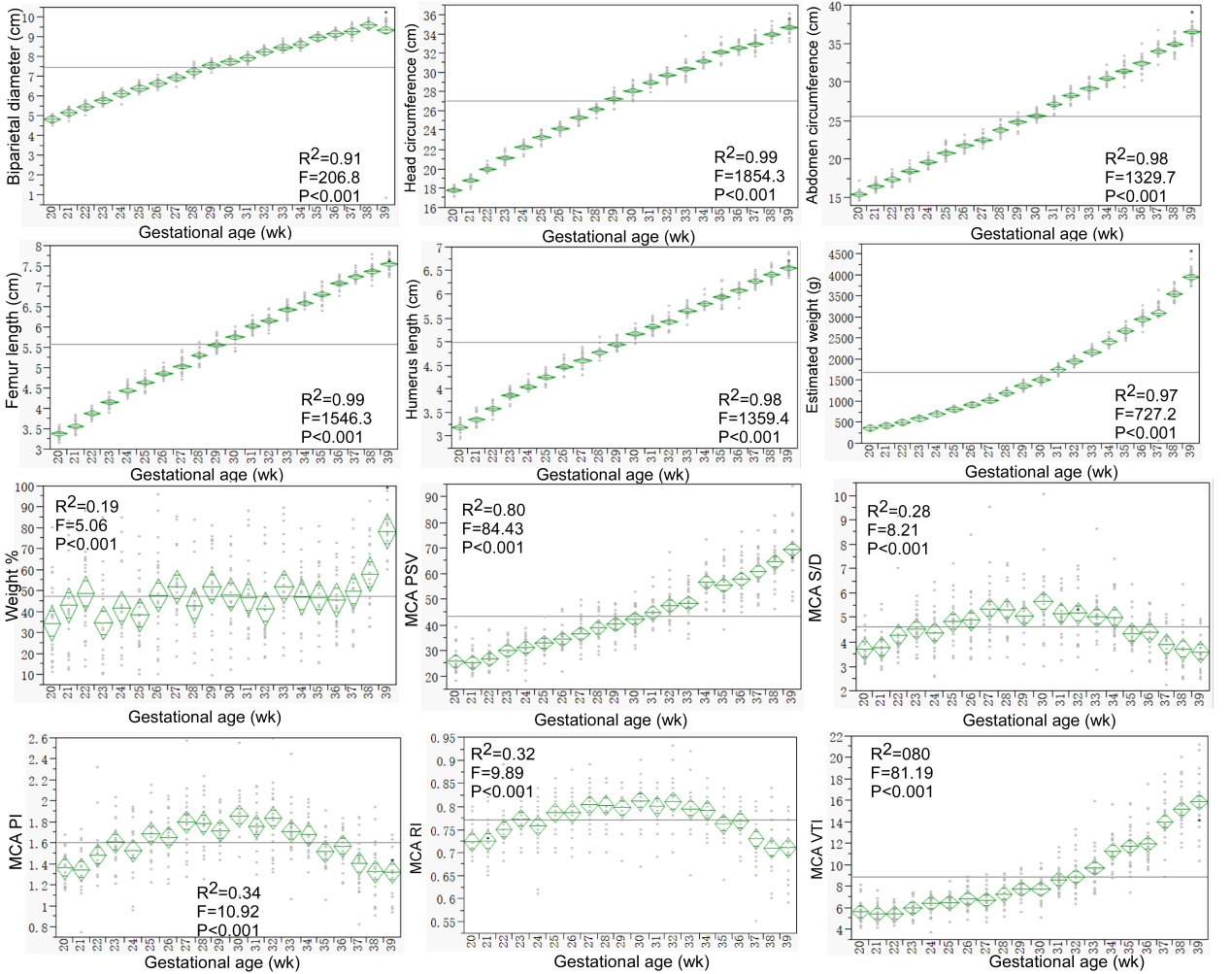


Supplemental Fig.1. Trends of data of fetal biology and middle cerebral artery (MCA) with gestational age. PSV, peak systolic velocity; S/D, PSV/EDV (end diastolic velocity); PI, pulsatility index; RI, resistance index; VTI, velocity time integral.


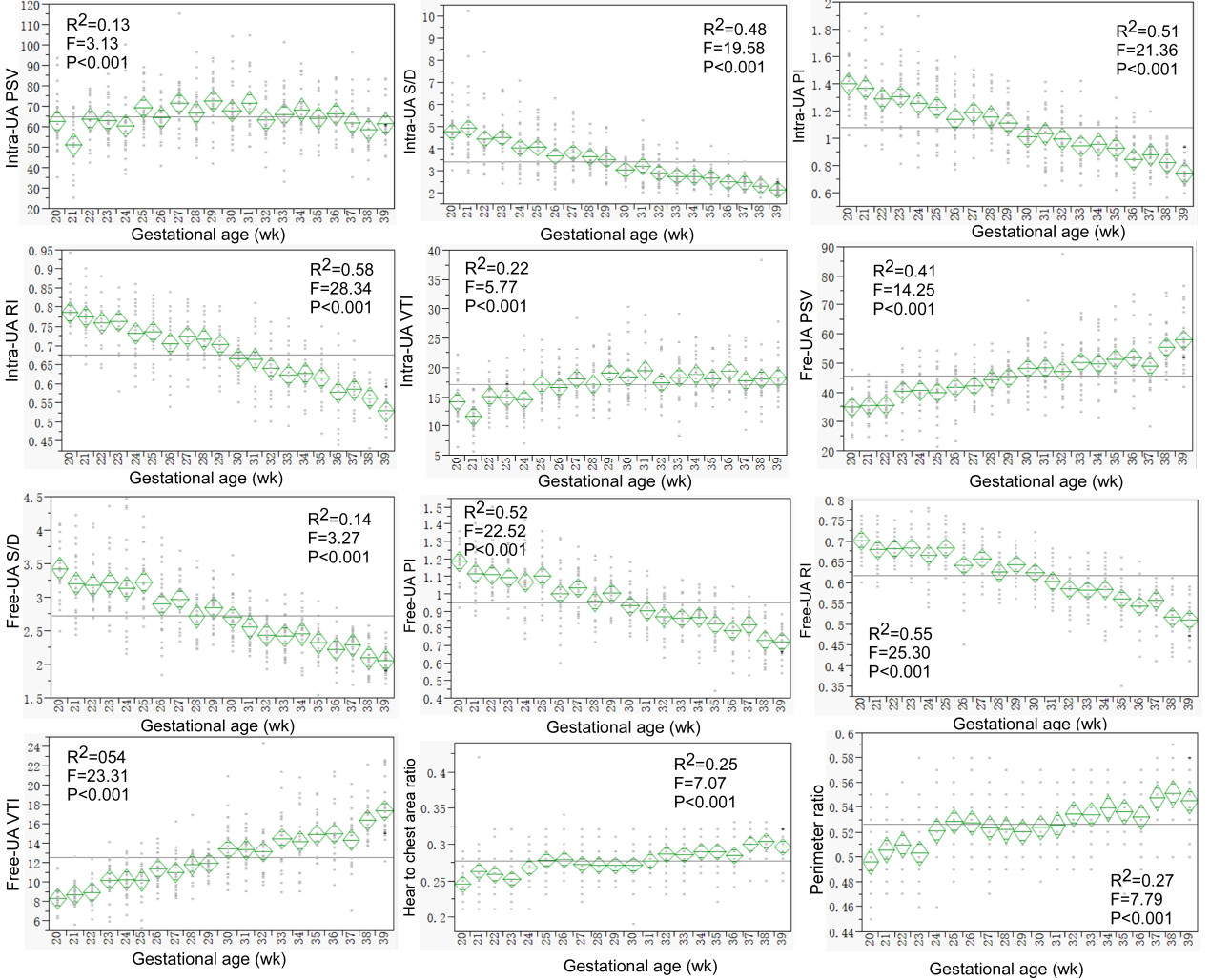


Supplemental Fig.2. Trends of data of Intra-abdominal umbilical artery (UA), free UA, and fetal heart and chest with gestational age. PSV, peak systolic velocity; S/D, PSV/EDV (end diastolic velocity); PI, pulsatility index; RI, resistance index; VTI, velocity time integral; Intra-UA, the UA segment inside the fetal abdomen; free-UA, the UA segment outside the fetal abdomen before the UA placental insertion.


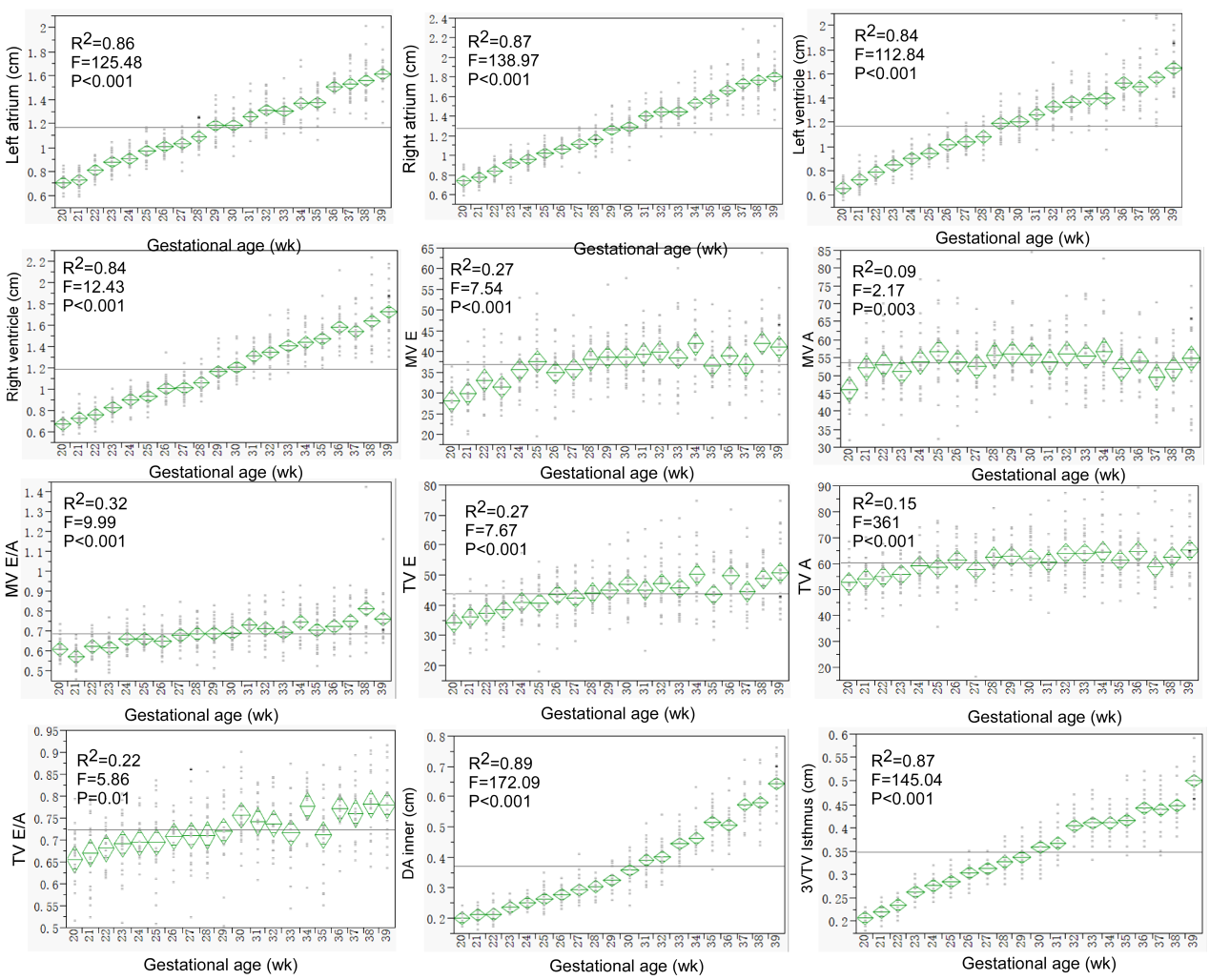


Supplemental Fig.3. Trends of the data of fetal heart, cardiac valves, ductus arteriosus (DA), and isthmus diameter at the vessel trachea view (3VTV) with gestational age. MV E, Mitral valve E-peak flow velocity; MV A, mitral valve A-peak flow velocity; TV E, tricuspid valve E peak; TV A, tricuspid valve A peak.
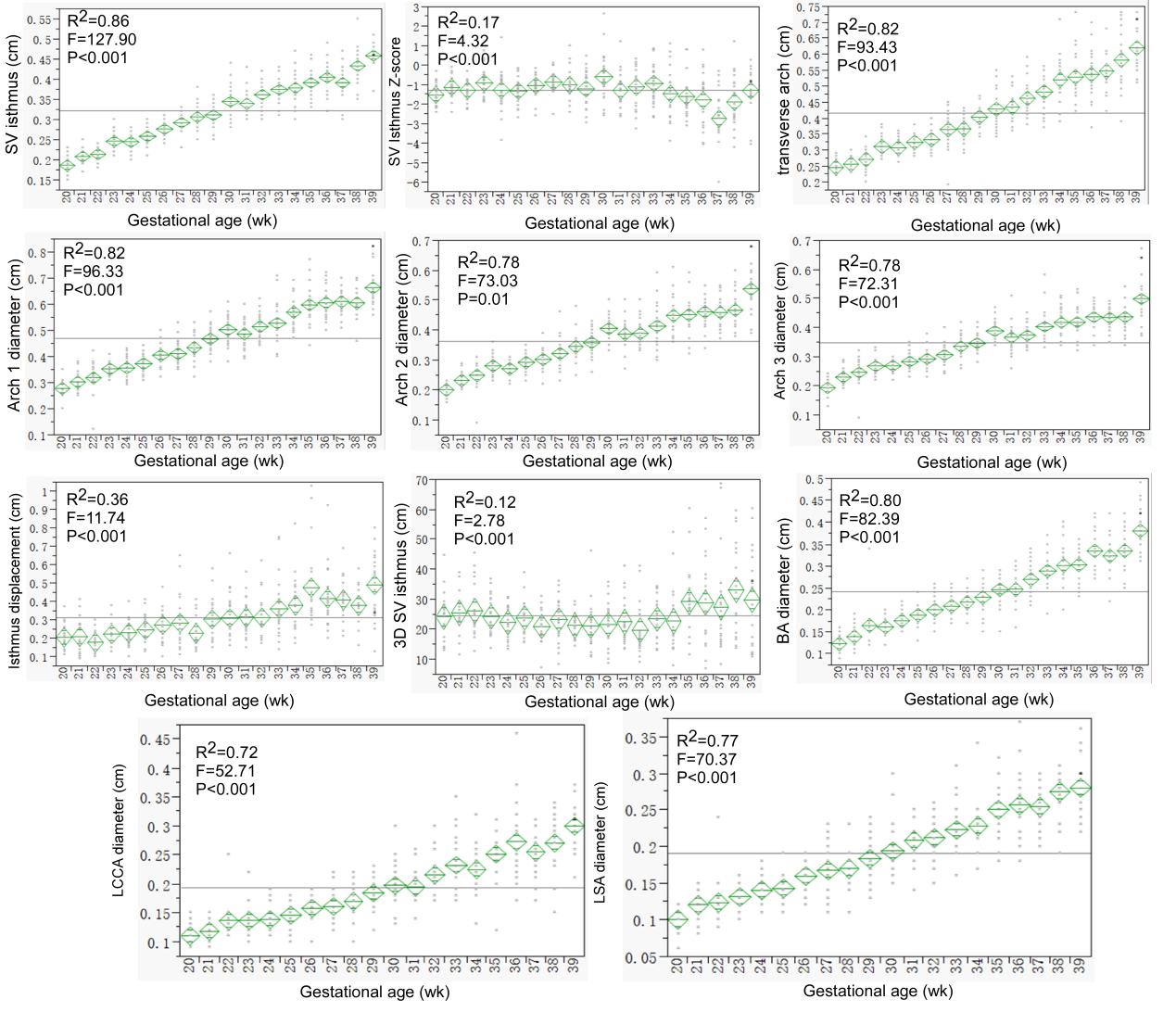


Supplemental Fig.4. Trends of data of isthmus, aortic arch, brachiocephalic artery (BA), left common carotid artery (LCCA), and left subclavian artery (LSA) with gestational age. SV, sagittal view; 3D, HD live flow image.


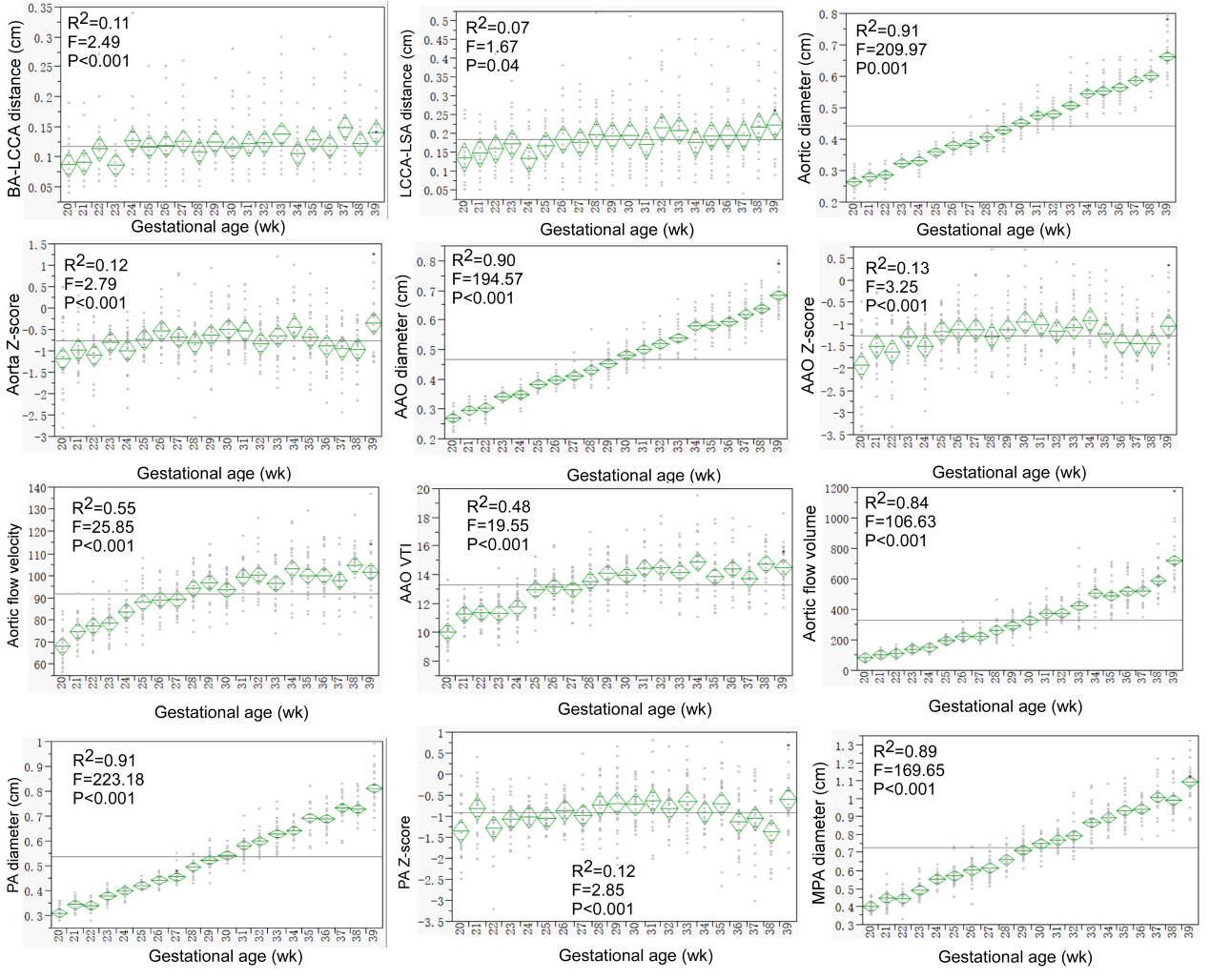


Supplemental Fig.5. Trends of arterial distance, diameter, Z score, flow velocity, volume, and VTI with gestational age. BA, brachiocephalic artery; LCCA, left common carotid artery; LSA, left subclavian artery; AAO, ascending aorta; VTI, velocity time integral; PA, pulmonary artery; MPA, main pulmonary artery.


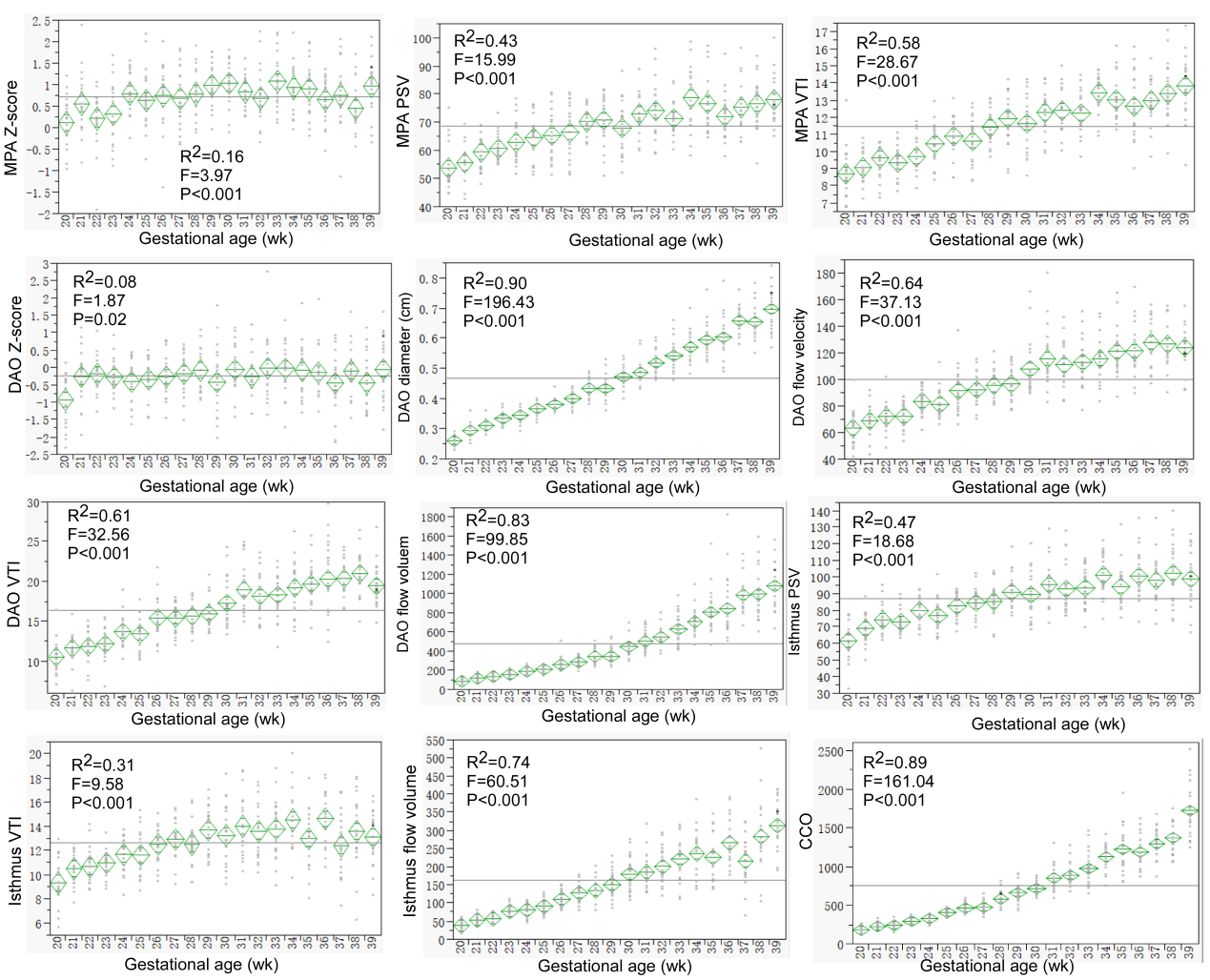


Supplemental Fig.6. Trends of main pulmonary artery (MPA), descending aorta (DAO), isthmus, and combined cardiac output (CCO) with gestational age. PSV, peak systolic velocity; VTI, velocity time integral.


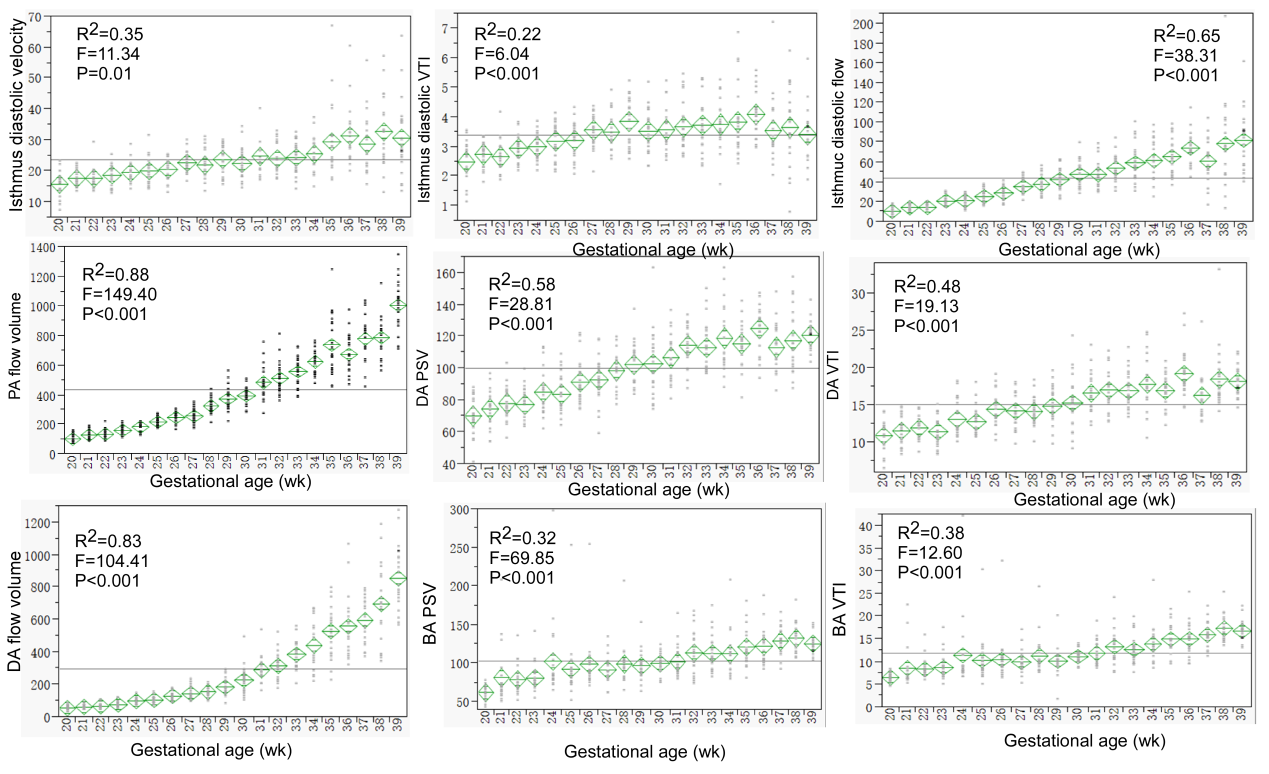


Supplemental Fig.7. Trends of the data of aortic isthmus, pulmonary artery (PA), ductus arteriosus (DA), and brachiocephalic artery trunk (BA). VTI, velocity time integral; PSV, peak systolic velocity.


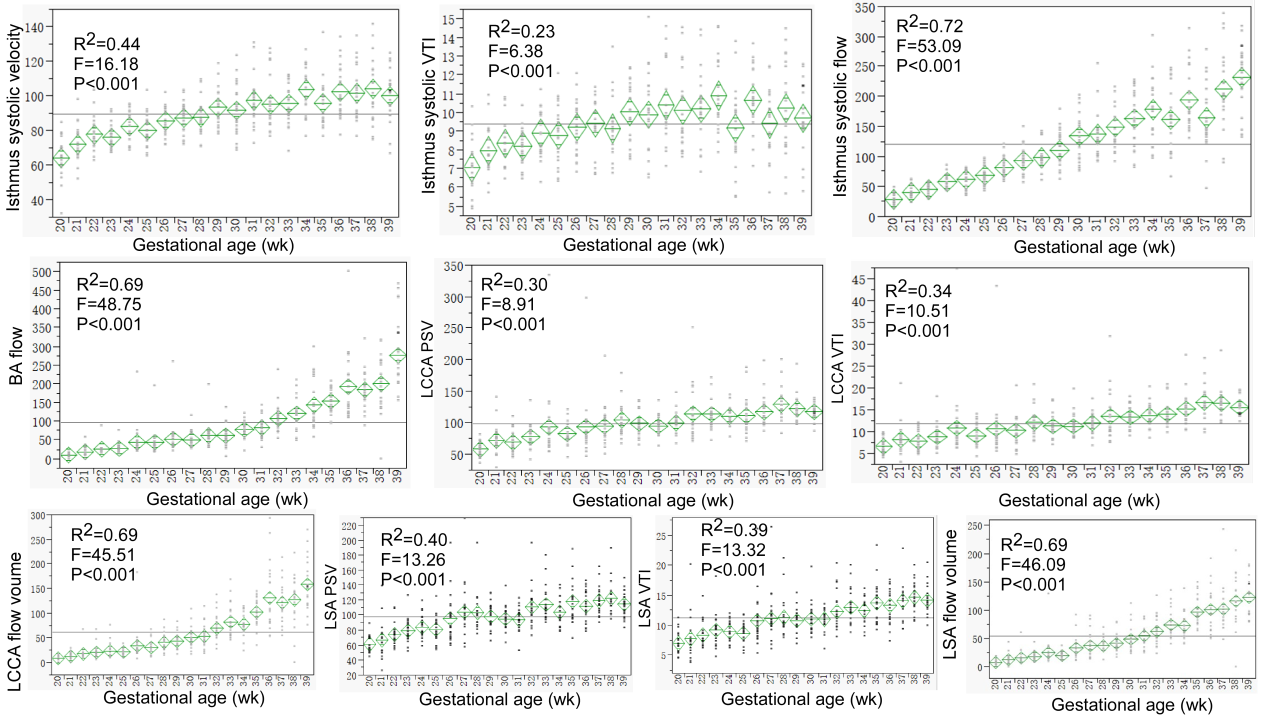


Supplemental Fig.8. Trends of the data of aortic isthmus, brachiocephalic artery trunk (BA), left common carotid artery (LCCA), and left subclavian artery (LSA) with gestational age. PSV, peak systolic velocity; VTI, velocity time integral.
